# Fibroadenoma versus phyllodes tumor: a vexing problem revisited!

**DOI:** 10.1186/s12885-020-07129-0

**Published:** 2020-07-13

**Authors:** Santosh Tummidi, Kanchan Kothari, Mona Agnihotri, Leena Naik, Prashant Sood

**Affiliations:** 1 Department of Pathology, All India Institute of Medical Sciences, Mangalagiri, 522503 Andhra Pradesh India; 2 Department of Pathology, Seth GSMC & KEMH, Parel, Mumbai, Maharashtra 400012 India; 3 Department of Pathology, LTMMC & LTMGH, Sion, Mumbai, 400012 India; 4 Department of Microbiology, All India Institute of Medical Sciences, Mangalagiri, 522503 Andhra Pradesh India

**Keywords:** Breast cytopathology, Fibroadenoma, Phyllodes tumor, Fine-needle aspiration

## Abstract

**Background:**

Fibroepithelial lesions of the breast include fibroadenoma (FA) and phyllodes tumor (PT). Fibroadenomas are benign while phyllodes tumor range from benign, indolent neoplasms to malignant tumors capable of distant metastasis. Our study was to determine the select cytologic features that can accurately distinguish FA from PT.

**Methods:**

A retrospective review was performed of patients who had histopathology follow up of FA or PT and on whom a pre-operative fine needle aspiration was performed. Cytologic criteria i.e. epithelial component, stromal component and background cellularity were assessed.

**Results:**

46 FA and 24 PT were specimens were reviewed. Median age and tumor size for FA and PT were 23.0 and 39.0 years, and 2.0 and 5.0 cm, respectively. Univariate analysis and regression models based on generalized estimating equations revealed that large opened out, folded epithelial sheets, frayed and irregular stromal fragment contours, spindle stromal cell nuclei, spindle cell nuclei in the background and background cell atypia are significant cytological predictors of PT. The GEE regression model achieved 78.9% diagnostic accuracy (*p* <  0.001) in identifying PT based on cytological features. Median epithelial: stromal ratio was 3.4 and 2.6 for FA and PT, respectively.

**Conclusion:**

Presence of large, opened out, folded epithelial sheets, frayed and irregular stromal contours with spindle nuclei, background spindle cells and atypia can help distinguish PT from FA.

## Background

Fibroepithelial lesions of the breast include fibroadenoma (FA) and phyllodes tumor (PT). Phyllodes tumors account for < 0.5% of all breast malignancies. They are characterized by a diverse range of biological behavior. The median age for PT is 45 years [1, 2]. PT can display locally destructive growth and even metastasize.

Phyllodes tumor resembles intracanalicular fibroadenoma at the benign end of the spectrum while malignant phyllodes tumor can be mistaken for primary breast sarcoma or sarcomatous carcinoma [1]. The distinction of phyllodes tumors from cellular/juvenile fibroadenomas is particularly challenging as the latter may show a cellular stroma. Presence of well-developed stromal fronds or exaggerated intracanalicular pattern of growth along with increased stromal cellularity on histology favour phyllodes tumor. There has been a close molecular relationship between fibroadenoma and phyllodes tumor demonstrated by the *MED12* mutations apart from the morphological resemblance (dimorphic pattern of epithelial and stromal components) [1, 3].

The preoperative diagnosis of PT is important for correct surgical planning to avoid a repeat surgery (at least 1 cm margin with wide local excision, has traditionally been the treatment of choice for phyllodes tumor). The cytodiagnosis of a high-grade malignant phyllodes tumor is not difficult as established in studies. However, the diagnosis of low-grade phyllodes tumor and its distinction from fibroadenoma on cytology is difficult due to overlapping features between the two lesions [1, 4]. Our study was done to evaluate the cytological features of phyllodes tumor with specific reference to certain cytological features that can help in differentiating it from fibroadenoma.

## Aims & objectives

To determine whether there are any significant differences between FA and PT with regards to cytological features and to correlate age, size, laterality in FA and PT.

## Material & Methods

We retrospectively reviewed 24 cases of phyllodes tumor and 46 cases of fibroadenoma (70 cases) diagnosed at our center. Only those cases were included in the study where the histopathological follow-up of the case was available for use as the gold standard for further analysis and comparison. In all cases, FNA was performed with a 23–25-gauge needle. A minimum of 2–3 passes were performed. Air-dried and alcohol-fixed smears were made and stained with Giemsa and Papanicolaou stains respectively. Overall cellularity of the smears was recorded as low, moderate or marked. The cytomorphology of the epithelial and stromal fragments, and the dispersed cell population in the background were studied. Features studied in the epithelial component were: number of clusters per 10 fields on a scanner (> 5 or < 5); nature of the cluster (staghorn, large folded opened out sheets, 3 dimensional); the degree of dissociation (mild/ moderate/marked at × 40); epithelial atypia (size in comparison to the size of small lymphocyte at high power, anisonucleosis, hyperchromasia: present or absent); mitosis; and apocrine metaplasia (present or absent).

Stromal component included number of stromal fragments per 10 fields on scanner (> 5 or < 5); size of fragments when viewed under scanner (small: < 50%, intermediate: 50–80%, large: > 80% of field); margins of stromal fragments (rounded/frayed/irregular); cellularity in stromal fragments under high power (mild/ moderate/marked); nature of the fragments (fibromyxoid/hyaline); traversing blood vessel (present or absent) and shape of the nucleus in stromal fragments under high power (spindle/oval).

For the dispersed cell population, the cellularity was expressed as mild, moderate or marked, and cell type as oval or spindle. The proportion of spindle cells (elongate cells with bipolar cytoplasmic projections) among dispersed cell population was recorded as < 10%, 10 – 30% and > 30% at high power. Dispersed cell atypia (present or absent) and mitosis (present or absent) were also noted. The presence of other cells in the background namely, cyst macrophages, columnar cells, giant cells, etc. were also recorded. Epithelial:stromal ratio was calculated by counting the total epithelial and total stromal fragments.

Since the cytological features for all FA and PT specimens were recorded by three blinded pathologists, these repeated measures were analyzed using generalized estimating equations (GEE). Following univariate GEE analysis, binary logistic GEE regression models were constructed to identify contextually relevant, independent cytological predictors that could reliably distinguish FA from PT. Variables showing quasi-separation were modeled using Firth’s bias reduction. Missing observations were not imputed. The diagnostic accuracy of the regression model was assessed by the area under the receiver operating characteristic curve (AUC). Non-repetitive patient characteristics were compared using Chi-square and Mann-Whitney U tests. A *p*-value < 0.05 was taken as significant. All analyses were conducted in SPSS v23.0.

## Results

This study was conducted in the Department of Cytopathology, of our Institute over a period of two years. A total of 70 cases were enrolled in this study, including 24 cases of phyllodes tumor and 46 cases with fibroadenomas, where the histopathological diagnosis of each case was available for use as the standard for further analysis. (Table 1).

**Table 1:** Comparison of non-repetitive patient characteristics between fibroadenoma and phyllodes tumour

**Characteristic**	**Fibroadenoma** (*n* = 46)	**Phyllodes tumor** (*n* = 24)	***p*value**
Age (years) (median, IQR)	23.0 (20.8–30.0)	39.0 (32.0–49.3)	**< 0.001**
Tumour size (cm) (median, IQR)	2.0 (1.4–2.3)	5.0 (3.3–7.0)	**< 0.001**
**Tumour location**			
Left centre	1 (2.2%)	1 (4.2%)	0.635
Left lower inner quadrant	3 (6.5%)	1 (4.2%)	0.687
Left lower outer quadrant	3 (6.5%)	2 (8.3%)	0.780
Left upper inner quadrant	4 (8.7%)	3 (12.5%)	0.615
Left upper outer quadrant	11 (23.9%)	9 (37.5%)	0.232
Right centre	0 (0.0%)	2 (8.3%)	**0.047**
Right lower inner quadrant	1 (2.2%)	0 (0.0%)	0.467
Right lower outer quadrant	6 (13.0%)	0 (0.0%)	0.064
Right upper inner quadrant	7 (15.2%)	1 (4.2%)	0.168
Right upper outer quadrant	10 (21.7%)	5 (20.8%)	0.930

*IQR* inter-quartile range

All patients were female. The most common age at presentation for FA was 21–30 years and 31–40 years for PT; with a median age of 23.0 and 39.0 years (*p* <  0.001), respectively. The youngest and oldest ages for FA were 16 and 45 years, respectively; and that for PT was 23 and 68 years, respectively. Left upper outer quadrant 11/46 (23.9%) was the commonest site involved with FA followed by the right upper outer quadrant 10/46 (21.7%). We had 15 left and 9 right-sided breast lumps of PT, of which left upper outer quadrant 9/24 (37.5%) was the commonest site involved followed by right upper outer quadrant 5/24 (20.8%) (Table 1).

**Fig. 1:**
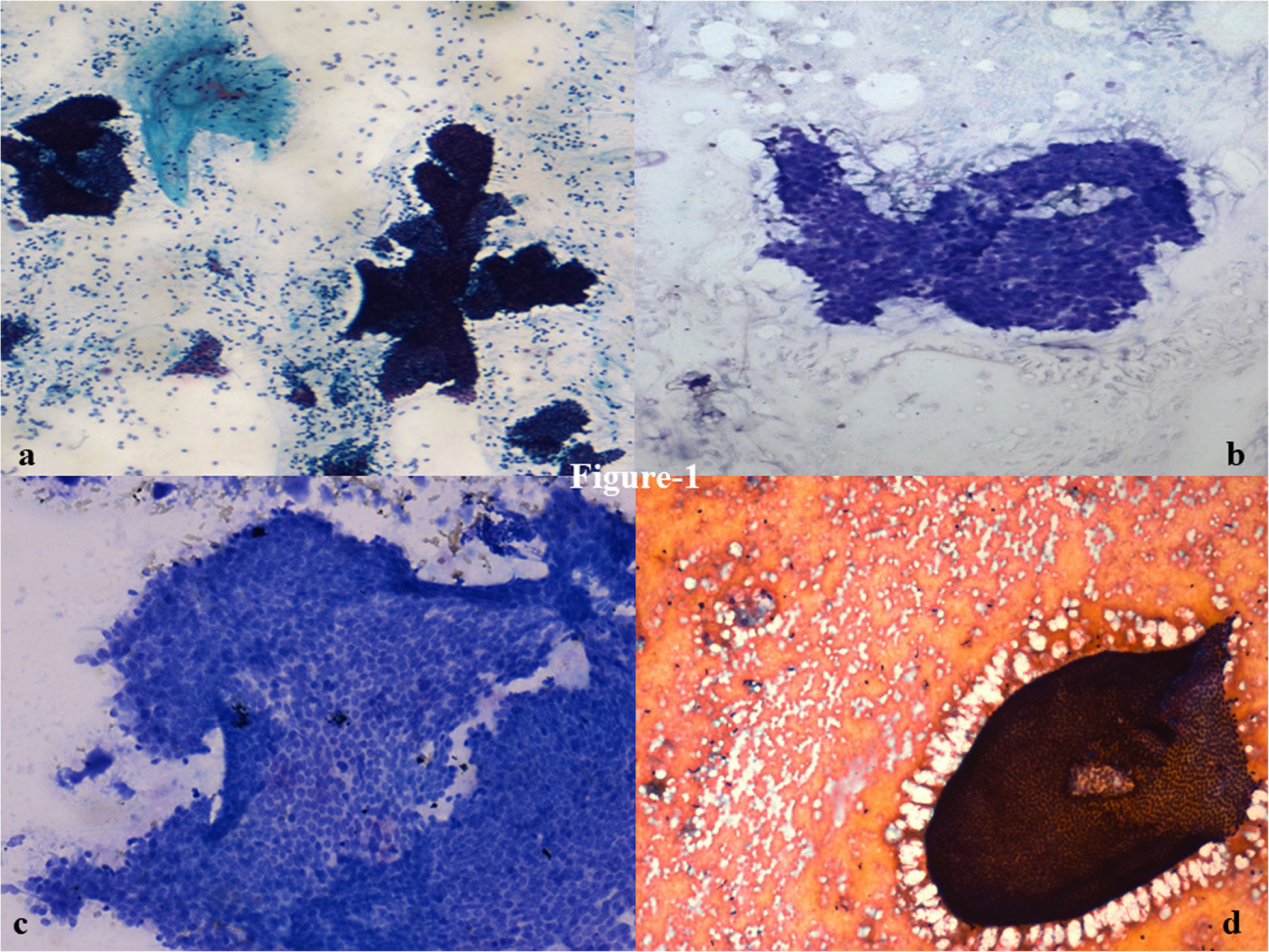
Cytosmears showing staghorn (**a**), folded (**b**), opened up sheet-like (**c**) and monolayered (**d**) ductal epithelial cells (× 100 & × 400, Giemsa, PAP)

Radiological tumor size was recorded for all tumors. The median size of fibroadenoma was 2.0 cm (interquartile range 1.4–2.3 cm), whereas phyllodes tumors had a median size of 5.0 cm (interquartile range: 3.3–7.0 cm) at presentation, making the latter significantly larger (*p* <  0.001) (Table 1). Overall cellularity was moderate to marked in 93% FA cases and 83% PT cases, with marked cellularity being less likely in PT (odds ratio [OR]: 0.28; *p* = 0.093). The number of epithelial cell clusters was significantly fewer in PT than FA (OR: 0.06; *p* = 0.019), with five or more clusters seen in 98% FA and only 38% PT cases. Large folded, opened out, epithelial sheets were much more common in PT (67%) than in FA (15%) (OR: 3.81; *p* <  0.001). In contrast, branched, staghorn, tubular and crowded clusters were more common in FA (Table 2) (Fig. 1). The remaining epithelial features did not show any significant differences between FA and PT. Epithelial cell dissociation was predominantly mild in 82% of FA and 87.5% of PT. Epithelial atypia was seen in 17 and 29% of FA and PT, respectively. Mitosis in epithelial cell clusters was seen in 20% of phyllodes tumors. Apocrine metaplasia was noted in 32 and 33% of FA and PT, respectively; and giant cells were seen in 32 and 42% FA and PT cases, respectively.

**Table 2:** Comparison of various cytological features which point towards phyllodes tumor versus fibroadenoma, based on univariate generalized estimating equations

**Cytological feature**	**Odds Ratio**	**95% CI of Odds Ratio**	***p*value**
**Epithelial component**			
Overall cellularity			
● Marked	0.28	0.06–1.24	0.093
● Moderate	0.59	0.16–2.27	0.448
● Mild^a^	0^a^	–	–
Epithelial clusters per slide			
● > 5	0.06	0.01–0.67	**0.019**
● 1–5	0.71	0.01–17.51	0.845
● < 1^a^	0^a^	–	–
Epithelial clusters			
● Branched	0.75	0.39–1.46	0.401
● Cohesive	1.23	0.42–3.60	0.705
● Opened large folded	3.81	1.86–7.82	**< 0.001**
● Staghorn	0.66	0.35–1.26	0.208
● Tubular	0.39	0.11–1.43	0.156
● Crowded	0.47	0.09–2.54	0.381
Degree of dissociation	1.28	0.22–7.37	0.779
Epithelial atypia	2.84	1.11–7.23	0.029
Epithelial mitosis	1.87	0.12–29.48	0.655
Apocrine metaplasia	0.66	0.25–1.70	0.386
Epithelial giant cells	0.74	0.29–1.85	0.516
**Stromal component**			
Fragments per slide			
● > 5	2.36	0.19–328.86	0.549
● 1–5	3.68	0.29–514.29	0.347
● < 1^a^	0^a^	–	–
Size of stromal clusters			
● Large	1.95	0.52–7.38	0.324
● Intermediate	1.74	0.62–4.89	0.294
● Small^a^	0^a^	–	–
Contours of stromal fragments			
● Irregular	3.29	1.03–10.54	**0.044**
● Frayed	4.85	1.07–21.88	**0.040**
● Round^a^	0^a^	–	–
Stromal cellularity			
● Marked	1.43	0.24–8.46	0.693
● Moderate	2.84	1.09–7.39	**0.032**
● Mild^a^	0^a^	–	–
Traversing blood vessels	1.75	0.76–4.02	0.189
Myxoid fragments	1.29	0.56–2.96	0.551
Fibro myxoid fragments^a^	0^a^	–	–
Shape of stromal nuclei			
● Spindle	3.35	1.31–8.55	**0.011**
● Plump	4.67	0.49–44.28	0.180
● Oval^a^	0^a^	–	–
**Background cell population**			
Background cellularity			
● Marked	0.75	0.19–2.65	0.604
● Moderate	1.10	0.40–3.07	0.851
● Mild^a^	0^a^	–	–
Shape of background cell nuclei			
● Spindle	7.93	3.79–16.58	**< 0.001**
● Plump	3.50	0.80–15.29	0.096
● Oval^a^	0^a^	–	–
Proportion of spindle cells			
● > 30%	399.67	22.70–737.93	**< 0.001**
● 10–30%	87.97	10.04–770.56	**< 0.001**
● < 10%^a^	0^a^	–	–
Background cell atypia	5.42	2.19–13.37	**< 0.001**
Background cell mitosis	9.93	0.94–105.30	0.057
**Other cells**			
Cyst macrophages	1.13	0.18–6.91	0.899
Columnar cells^a^	0^a^	–	–

^a^Reference category

**Fig. 2:**
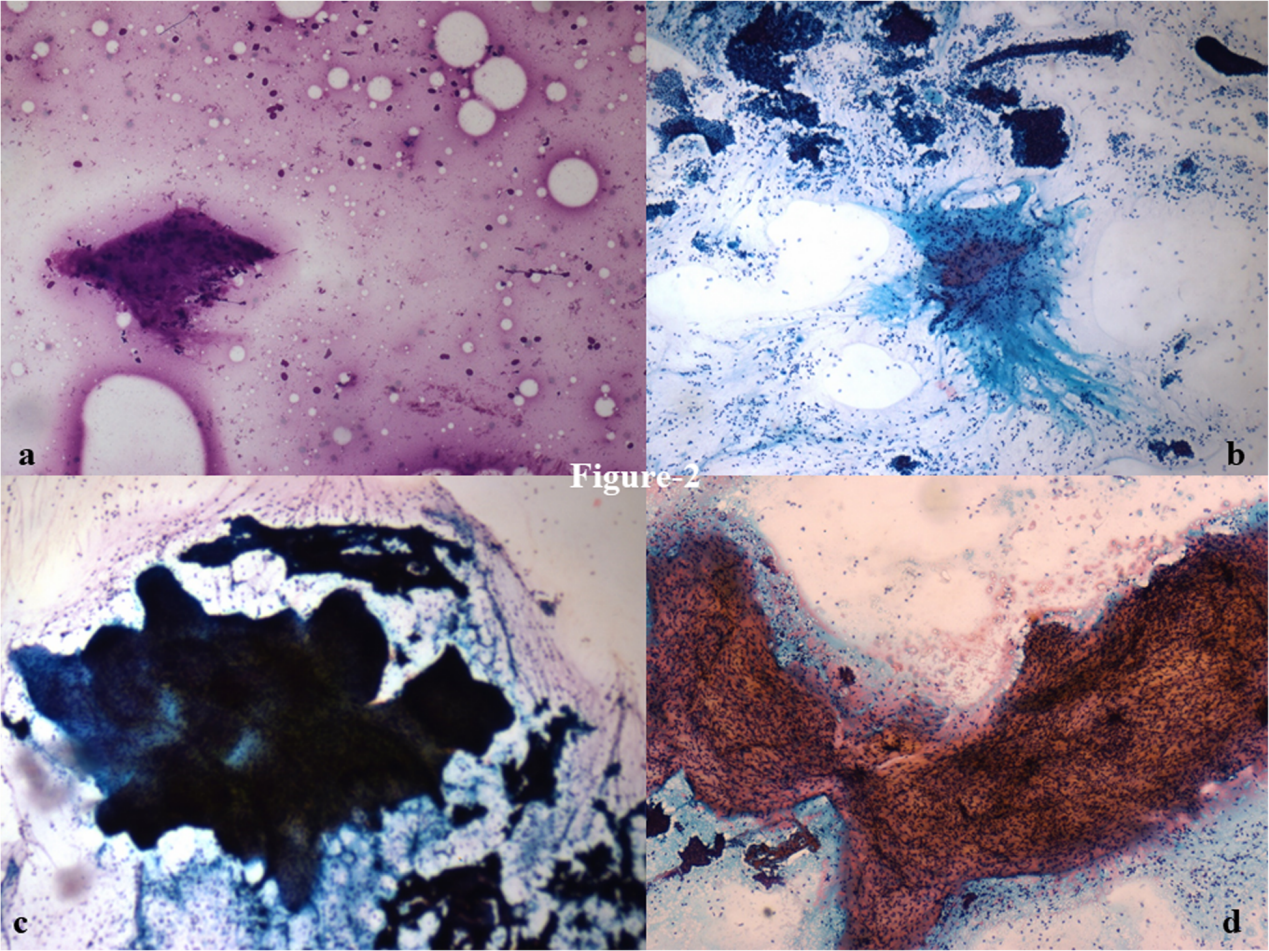
Cytosmears showing (**a**) small (< 50% field), (**b**) moderate (50–80% field) & (**c, d**) large (> 80% field) sized stromal fragments with fraying. (× 100, Giemsa & PAP)

**Fig. 3:**
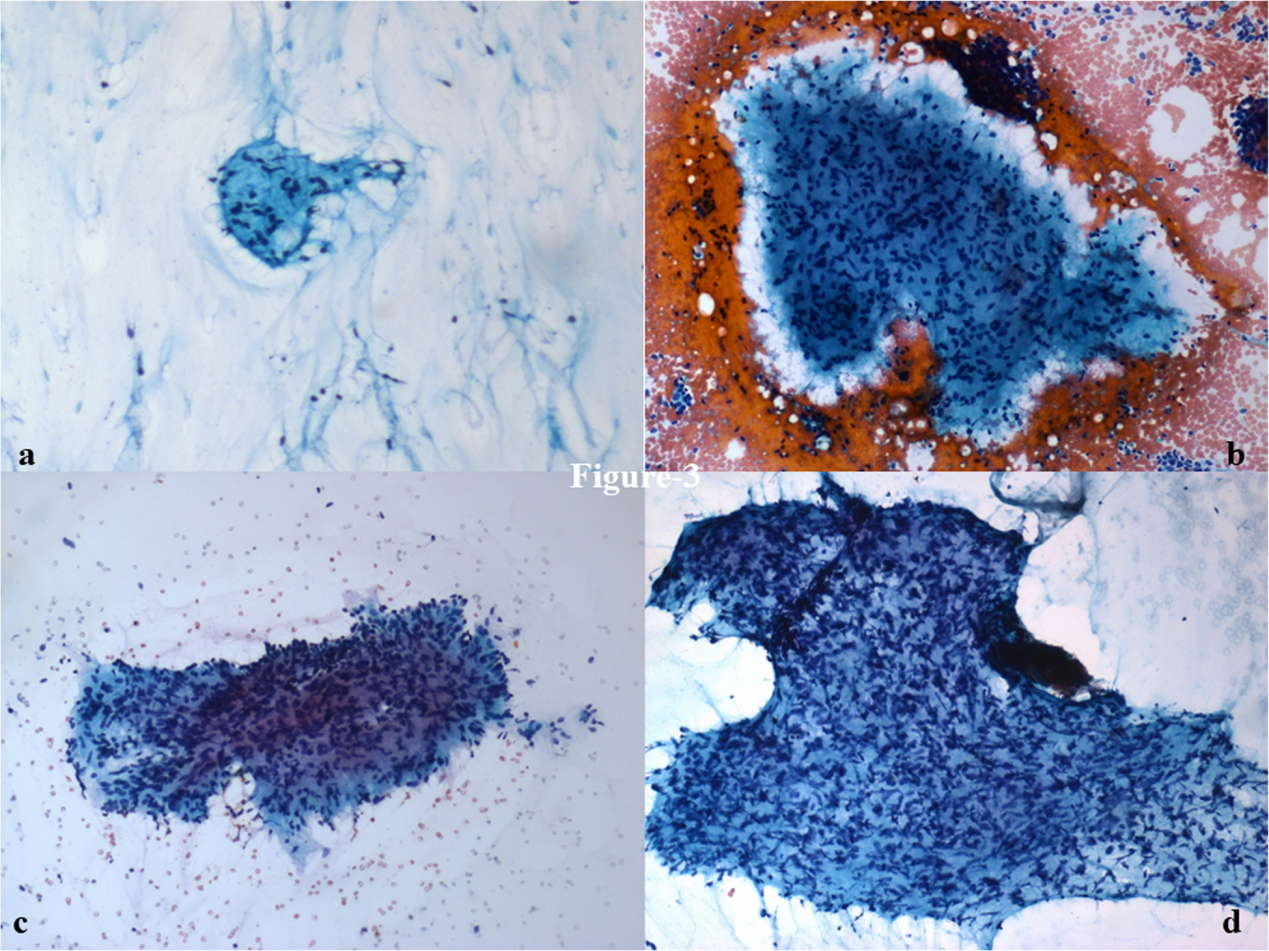
Cytosmears showing (**a**) mild, (**b**) moderate, and (**c, d**) marked stromal cellularity. (× 100, PAP)

**Fig. 4:**
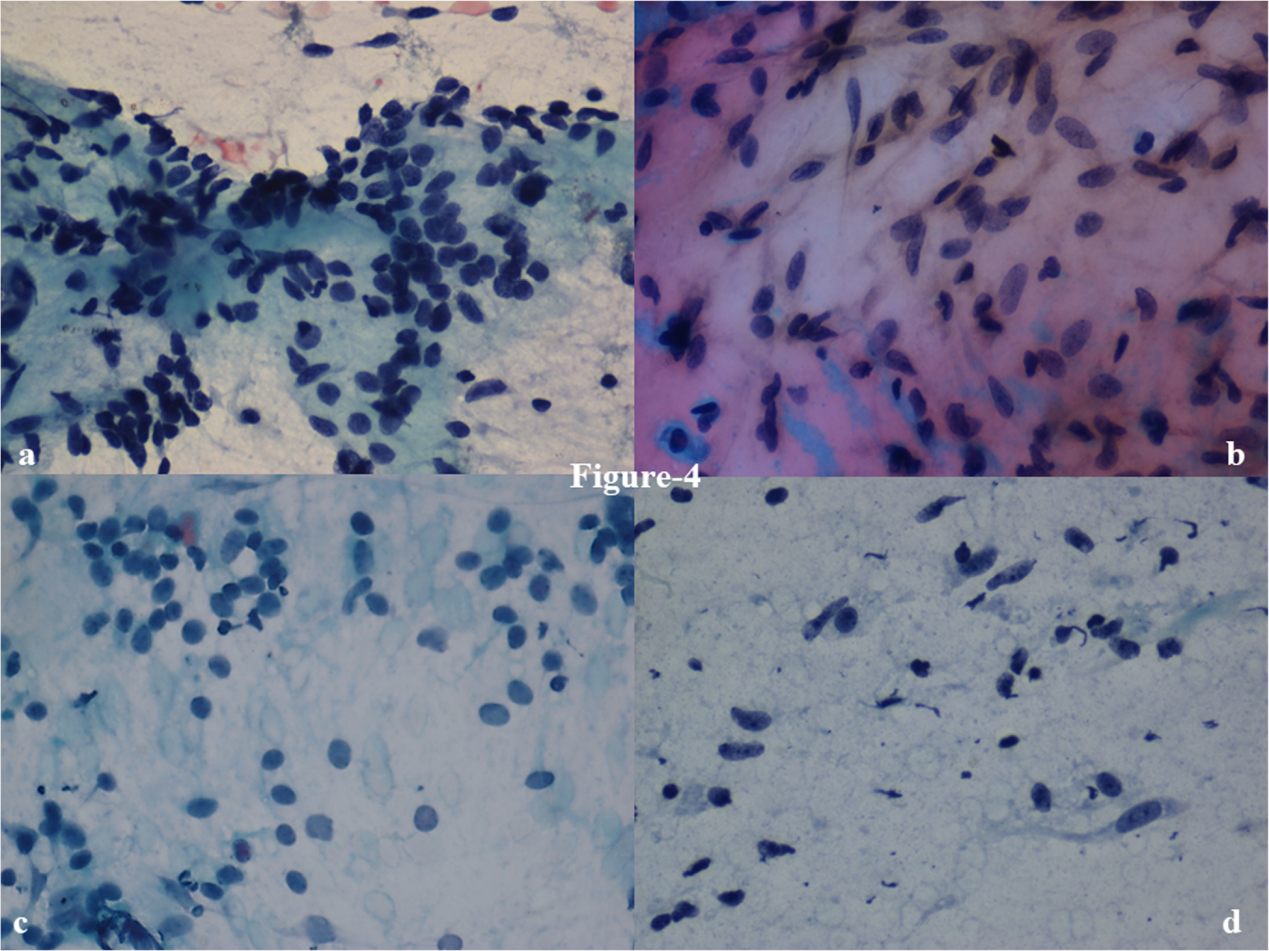
Stromal fragments matrix showing (**a**) plump oval-shaped cells - fibroadenoma, and (**b**) spindle cells - phyllodes tumor, respectively. Also scattered in the background are similar (**c**) oval cells of FA and (**d**) spindle cells with bipolar cytoplasmic projections of PT. (× 400, PAP)

Among the stromal fragments, five or more fragments were more commonly seen in PT (75%) than in FA (54%). There was one case of fibroadenoma that had ≤ 1 stromal fragment. Small stromal fragments were more common in FA (61%) as compared to PT (42%). On the other hand, intermediate to large stromal fragments were more common in PT (58%) than in FA (37%) (Fig. 2). Stromal fragments were significantly more frayed (OR: 4.85; *p* = 0.040) and irregular (OR: 3.29; *p* = 0.044) in phyllodes tumor (83%) as compared to fibroadenoma (52%) (Table 2). Cellularity of stromal fragments was moderate (OR: 4.85; *p* = 0.032) to marked (OR: 1.43; *p* = 0.693) in PT (58%) as compared to FA (28%) (Table 2) (Fig. 3). Traversing blood vessels were noted in 33% FA and 46% PT cases. 89% of FA had fibromyxoid fragments with 52% of these having spindle cells within them. In contrast, 88% of phyllodes tumors had fibromyxoid fragments with 92% of these with a predominance of spindle cells (OR: 3.35; *p* = 0.011) (Table 2) (Fig. 4a, b). The epithelial to stromal ratio was 2.6 and 3.4 for phyllodes and fibroadenoma, respectively; with an overall stromal:epithelial ratio across both groups being 4.5:1.

**Fig. 5:**
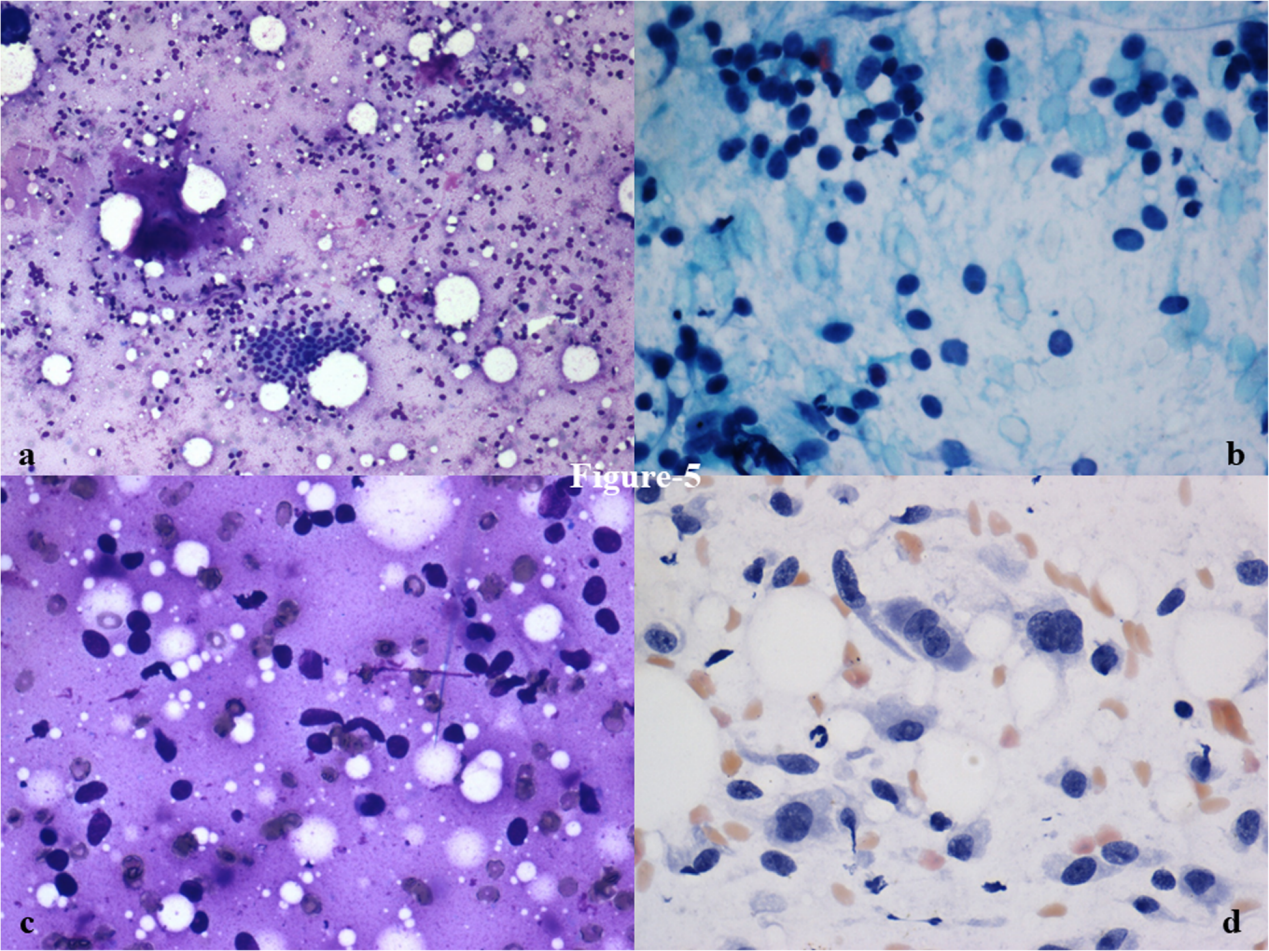
Cytosmears showing the gradient from predominantly oval cells (**a**) scattered in background to (**b**) < 10% spindle cells, (**c**)10–30% spindle cells, and (**d**) > 30% spindle cells with nuclear atypia (> 2 times the size of small lymphocyte). (× 100 & × 400, Giemsa & PAP)

Background cellularity was moderate to marked in 76% of FA as compared to 83% of PT. A significantly higher number of background spindle cells (ranging between 10 and 30%) were seen in phyllodes tumor (63%) as compared to fibroadenoma (13%), while > 30% spindle cells were seen in 33% of PT and 2% of FA cases, respectively (*p* <  0.001) (Fig. 5). Background cellular atypia was also significantly higher in PT (50%) than in FA (11%) (OR: 5.42; *p* <  0.001) (Table 2). 50% of FA and 58% of PT had cyst macrophages in the background.

To ascertain which of the above cytology features could be employed as independent predictors for distinguishing PT from FA, a GEE binary logistic regression model was constructed (Table 3). The model revealed that the presence of large folded, opened out epithelial sheets; frayed stromal fragments; and spindle cells and atypia in dispersed background cells significantly increased the odds of a tumor being phyllodes. In contrast, the presence of marked overall epithelial cellularity with oval stromal nuclei predicts lower chances of the tumor being phyllodes. The regression model was able to predict PT accurately in 78.9% cases (AUC: 0.79; 95% confidence interval: 0.72–0.86; *p* <  0.001).

**Table 3:** Best fitting generalized estimating equations type III model predicting the diagnosis of phyllodes tumor as compared to fibroadenoma

**Cytological feature**	**Odds Ratio**	**95% CI of Odds Ratio**	***p*value**
Marked epithelial cellularity	0.16	0.22–1.12	0.064
Large opened epithelial sheets	3.41	1.51–7.68	0.003
Frayed stromal fragment contours	3.89	0.99–15.14	0.050
Oval stromal nuclei	0.31	0.12–0.77	0.012
Spindle background cell nuclei	7.14	2.85–17.92	< 0.001
Background cell atypia	7.15	2.29–22.28	0.001

Cytohistological correlation was done. Out of the 46 cases labeled fibroadenoma on cytology, 45 were concordant on histopathology and one case showed features of benign phyllodes tumor. Among the 24 cases labeled as phyllodes tumor on cytology, 17 were benign phyllodes, 03 borderline phyllodes and 3 were malignant. All the three malignant phyllodes had been diagnosed as malignant on cytology. One benign phyllodes tumor was discordant on histopathology and was reported as a fibroadenoma.

The two discordant cases were reviewed. In the case of benign PT misdiagnosed as FA, the patient was a 32-year-old lady with a 3 cm lump and the likely cause for the discrepancy was low overall cellularity with very few, small stromal fragments. On review, it was noted that a possible clue was that all the epithelial fragments were in the form of opened out sheets and a few spindle cells were seen in the background. Thus, although a definite diagnosis of PT was not possible, benign fibroepithelial lesion would have been a more appropriate diagnosis. The case of FA misdiagnosed as benign PT was a 23-year-old patient with a 7 cm lump which on aspiration had shown > 30% dissociate spindle cells in the background.

## Discussion

Phyllodes tumor (PT) of the breast was first described by Chelius in 1827 and was later termed as *cystosarcoma phyllodes* by Johannes Muller in 1838. The use of the term “cystosarcoma” was intended to describe the cystic and fleshy appearance [5, 6]. Despite extended historic existence of phyllodes tumor of the breast, classification is still not ideal; there is difficulty in distinguishing it from fibroadenoma at the benign end of the spectrum, and problems subdividing the 3 recognized grades of phyllodes tumors. There is also a small proportion of benign fibroepithelial neoplasms that histologically do not fit into the category of fibroadenoma or phyllodes tumor [7–11].

Phyllodes tumor can range from benign to malignant. The classification of PT by the World Health Organization (WHO) into benign, borderline, and malignant is based upon a combination of several histologic features including stromal cellularity, mitotic activity, nuclear atypia, tumor margin appearance, and stromal overgrowth [1, 12]. The majority of PT are benign in nature. The risk of local recurrence can range from 27% in malignant PT to 17% in benign PT. 22% of malignant PTs may have distance metastasis [1, 10].

There are no well-defined criteria or clear-cut offs for individual histologic parameters. Approximately 15% of the cases can be misdiagnosed. Difficulty is experienced even during histology, where benign phyllodes tumors may resemble intracanalicular FA; with the presence of intraductal leaf-like stromal growth being a characteristic feature in the former condition. Neither clinical examination nor radiological appearances can differentiate these two tumors. Juvenile/cellular fibroadenomas are notorious for adding to the diagnostic dilemma owing to their increased stromal cellularity. Hence, the diagnosis of PT has remained a challenge, particularly in the distinction of benign PT from fibroadenoma [3, 5, 7, 13–16]. There is almost 100% sensitivity of FNA in the diagnosis of breast carcinoma when combined with radio mammography and clinical correlation [17]. However; the reported sensitivity of FNA in the diagnosis of phyllodes tumors is reported to range from 32 to 77% [18, 19]. The poor sensitivity of FNA is mainly because of the failure to detect phyllodes tumors (benign and borderline) on FNA smears. The benign and borderline PT represents 80% of all phyllodes tumor and are mistakenly diagnosed as fibroadenoma. Malignant phyllodes tumors diagnosis is usually straight forward and is not much of a problem when the stromal component is the sole or dominant one with pleomorphism, marked atypia, and high-mitotic activity [1]. However, fine needle aspiration cytology cannot distinguish between benign and borderline PT but this distinction is not vital pre-operatively.

Our study had a total of 70 cases which included 46 fibroadenoma and 24 phyllodes cases drawn over a period of 2 years (Table 4). Fibroadenomas have been reported in patients younger than 30 years of age, whereas phyllodes tumor are more common in older patients, usually between the age group of 40 and 50 years [21]. Our patients had a median age of 39.0 years for PT which was similar to that seen in studies by Demian et al. (40 years) [22], Veneti et al. (42.2 years) [20] and Maritz et al. (44 years) [23]. None of our cases of PT were below the age of 20 years. The age of the patient can thus be helpful while evaluating a cellular fibroepithelial lesion.

**Table 4:** Comparison of number of cases in each group, total cases and duration of study with other studies

	**Bhattarai et al 2000** [9]	**Krishnamurthy et al 2000** [8]	**Scolyer et al 2001** [29]	**Veneti et al 2001** [20]	**Jayaram et al 2002** [19]	**Badhe et al 2002** [11]	**El Hag et al 2010** [17]	**Bandyopadhya et al 2010** [2]	**Maritz et al 2017** [23]	**Present study**
Duration of study	15 years	–	8 years	8 years	7 years	3 years	6 years	3 year	10 years	**2 years**
PT/FA	57/23	12/33	8/13	18/18	28/00	9/9	15/12	10/25	17/50	**24/46**
Total	80	45	21	36	28	18	27	35	67	**70**

Left breast lumps were the most common site of involvement in our study which was in concordance with the results of Maritz et al. [23]. Epithelial features (number of fragments, atypia, apocrine metaplasia, dissociation, and mitotic activity) did not show significant difference between fibroadenoma and phyllodes tumors in studies done by Deen et al. [24], Krishnamurty et al. [8], Bandyopadhayay et al. [2] (Table 5). Similar findings were noted in our study but the presence of large opened out, folded epithelial fragments was statistically significant in phyllodes tumor. This most likely represents the epithelium of the predominant exaggerated intracanalicular proliferation [25, 26].

**Table 5:** Comparison of epithelial, stromal and background cellularity among our study and other researchers

	**PT%/FA%**	**PT%/FA%**	**PT%/FA%**	**PT%/FA%**	**PT%/FA%**	**PT%/FA%**	**PT%/FA%**	**PT%/FA%**
**Epithelial component**	**Krishnamurthy et al 2000 **[8]	**Veneti et al 2001 **[20]	**Scolyer et al 2001** [29]	**Shimizu et al 2002** [15]	**El Hag et al 2010 **[17]	**Bandyopadhy et al 2010** [2]	**Maritz et al 2017** [23]	**Present study**
Overall cellularity (Mod-marked)	33/30	83/83	87/100	78/100	87/100	83/78	–	83/93
No. of fragments (> 5)	83/94	55/33	87/100	78/100	80/100	80/92	23/95	38/98
Staghorn; branched; open monolayer	-- 70/67	42/78 65/30	-/93 85/75	39/56 78/10	-- --	22/75 60/25	-- --	**58/78** **67/15**
Atypia	–	17/05	00/00	–	00/00	00/00	–	29/17
Mitosis	–	–	–	–	00/00	00/00	–	33/00
**Stromal component**	**Krishnamurthy et al 2000** [8]	**Scolyer et al 2001** [29]	**Veneti et al 2001** [20]	**Badhe et al 2002** [11]	**El Hag 2010** [17]	**Bandyopadhy et al 2010** [2]	**Maritz et al 2017** [23]	**Present study 2018**
Stromal fragments (> 5)	33/27	–	39/28	78/20	67/25	70/44	70/45	75/54
Stromal size (Inter-large)	20/41	75/69	39/28	70/32	67/ 25	60/32	80/30	58/38
Stromal cellularity Mod-marked	93/60	62/15	28/40	100/33	–	90/20	82/40	58/28
Traversing blood vessel	–	25/23	–	55/22	–	30/20	25/23	45/32
Type of nucleus (spindle)	75/15	62/00	85/27	–	93/00	80/10	41/14	**92/52**
Frayed margin	64/39	–	–	72/40	93/66	40/72	60/40	**83/52**
Fibro myxoid fragments	–	–	–	–	100/67	70/−	–	88/89
**SER**	**Bhattarai et al 2000** [9]	**Jayaram et al 2001** [19]	**Badhe et al 2002** [11]	**Bandyopadhy et al 2010** [2]	**Maritz et al 2017** [23]	**Present study **		
Stromal-epithelial ratio	7.6:1	6:1	> 1:0.5	> 1:1	5.2:1	4.5:1		
**Background cells**	**Krishnamurthy et al 2000** [8]	**Badhe et al 2002** [11]	**El Hag et al 2010** [17]	**Bandyopadhy et al 2010** [2]	**Maritz et al 2017** [23]	**Present study **		
Cellularity	58/84	77/88	73/92	70/68	–	83/76		
**Spindle (> 10%)**	**75/21**	**100/33**	**100/10**	**90/16**	**47/20**	**96/15**		
Atypia	16/00	–	47/00	–	12/00	50/11		
Mitosis	08/00	–	07/00	–	–	08/00		
Cyst macrophages	–	11/48	90/50	–	–	60/50		
Giant cells	–	00/42	73/25	10/00	–	42/32		
Apocrine metaplasia	17/12	11/50	07/25	10/08	–	33/32		

Studies done by Veneti et al. [20], Jayaram et al. [19], Bandyopadhay et al. [2] and El Hag et al. [17] had found that presence of an increased number of stromal fragments with hypercellularity, larger size and higher stromal to epithelial (S:E) ratio favor phyllodes (Table 5). Background dispersed cells showed a significant difference between FA and PT. There was moderate to marked background cellularity in 84%, percentage of spindle cells > 10% in 96% cases, and cytologic atypia with mitosis in 50% cases of phyllodes tumor. The proportion of spindle cells as a cut-off for malignancy was > 30% in studies done by Krishnamurthy et al. [7] and Maritz et al. [23] whereas the same was > 10% in the study by El Hag et al. [17] (Table 5). Presence of long spindle cells > 30%, fibroblastic pavements and spindle nuclei in stroma is considered a diagnostic triad for phyllodes tumor [17]. Spindle cells may be noted in FA (cellular variant) but they generally do not exceed 30% of the total dispersed cell population. Presence of cyst macrophages, columnar cells and apocrine metaplasia did not show any significant difference between FA and PT; similar findings were found in studies by Bhattarai et al. [8], Deen et al. [24], and Dusenbery et al. [5], hence proving to be of little value in distinguishing the two lesions.

Distinction of cellular fibroadenoma and benign phyllodes tumor has been attempted by Tay et al. [25], Ross et al. [26], and Faiz et al. [27]. All these histopathological studies had found that fibroadenomas in the pediatric age group tend to have increased stromal cellularity and should be interpreted with caution. Even the mean mitotic counts could be up to 7 mitosis/10 HPF in both the conditions. The age of patients and stromal fronds along hypercellular stroma can be diagnostic clues. The term benign fibroepithelial lesion may be used for cases where a clear diagnostic distinction cannot be made, although the term should be used sparingly [1, 28, 29]. Features favoring phyllodes tumor over fibroadenoma in biopsy include the tumor size (> 3 cm), mitosis > 3/10 HPF, stromal overgrowth, stromal fragmentation, infiltration into fat, stromal heterogenicity and stromal pleomorphism [30].

Phyllodes tumor has traditionally been excised with wide tumor-free margins, and some authors have suggested a margin of at least 1 cm [31, 32]. Recent studies however suggest that a conservative approach could be accorded to benign phyllodes tumors that have been initially enucleated without margins, as the recurrence rate of benign fibroepithelial lesions is low and not associated with original margin status. Recurrent and malignant phyllodes tumors require excision with negative margins [28, 32].

The accuracy of cytologic diagnosis of fibroepithelial tumors can be improved by applying a semi-quantitative assessment utilizing selected criteria [33, 34]. Our study was one such attempt to apply semi-quantitative criteria to identify subtle differences between fibroadenoma and phyllodes tumor. These criteria will be helpful while reporting cases in the grey zone. Cellular spindle cell stromal fragments and increased background spindle cells along with large, opened out epithelial sheets favor phyllodes, evidence that is reinforced by our regression model. The lack of a cohort assessed by core needle biopsies and its comparison with FNA remains a limitation of our study, especially with the former being increasingly recommended [35, 36]. However, the goal of our study was to unravel FNA findings which can help distinguish PT from FA in resource-limited settings where core needle biopsies cannot be afforded by patients due to financial constraints. Our study successfully identified such FNA features despite its limitations.

## Conclusion

Although core biopsies are replacing fine needle aspirations in many settings, FNA continues to be used for palpable breast lesions in patients with financial constraints. Our study describes FNA features that can help distinguish PT from FA in such resource-limited settings. Stromal features (frayed irregular borders, increased stromal cellularity with a predominance of spindle cells), increased background spindle cells, and predominance of large folded, opened out epithelial sheets are the features to look for while distinguishing these two entities.

## Data Availability

The datasets used and/or analysed during the current study available from the corresponding author on reasonable request.

## Abbreviations

FA: Fibroadenoma
FNAC: Fine needle aspiration cytology
PT: Phyllodes tumor
S:E: Stromal to epithelial
WHO: World Health Organization
